# The new and recurrent FLT3 juxtamembrane deletion mutation shows a dominant negative effect on the wild-type FLT3 receptor

**DOI:** 10.1038/srep28032

**Published:** 2016-06-27

**Authors:** Nadine Sandhöfer, Julia Bauer, Katrin Reiter, Annika Dufour, Maja Rothenberg, Nikola P. Konstandin, Evelyn Zellmeier, Belay Tizazu, Philipp A. Greif, Klaus H. Metzeler, Wolfgang Hiddemann, Harald Polzer, Karsten Spiekermann

**Affiliations:** 1 Department of Internal Medicine III, University Hospital Grosshadern, Ludwig-Maximilians-University (LMU), Munich, Germany; 2 German Cancer Consortium (DKTK), Heidelberg, Germany; 3 German Cancer Research Center (DKFZ), Heidelberg, Germany

## Abstract

In acute myeloid leukemia (AML), the Fms-like tyrosine kinase 3 (*FLT3)* is one of the most frequently mutated genes. Recently, a new and recurrent juxtamembrane deletion mutation (p.Q569Vfs*2) resulting in a truncated receptor was identified. The mutated receptor is expressed on the cell surface and still binds its ligand but loses the ability to activate ERK signaling. FLT3 p.Q569fs-expressing Ba/F3 cells show no proliferation after ligand stimulation. Furthermore, coexpressed with the FLT3 wild-type (WT) receptor, the truncated receptor suppresses stimulation and activation of the WT receptor. Thus, FLT3 p.Q569Vfs*2, to our knowledge, is the first *FLT3* mutation with a dominant negative effect on the WT receptor.

The Fms-like tyrosine kinase 3 (*FLT3)* gene encodes a receptor tyrosine kinase (RTK), which is mostly expressed on hematopoietic progenitor cells and enables these cells to proliferate and differentiate. The high prevalence of activating mutations of the *FLT3* gene in acute myeloid leukemia (AML) indicates the importance of *FLT3* for physiological hematopoiesis^1^,^2^. The most common alterations occur in two functional domains of the receptor. Internal tandem duplications (ITD) disrupt the autoinhibitory function of the juxtamembrane domain and convey ligand-independent phosphorylation and activation of FLT3^3^. Point mutations within the activation loop of the tyrosine kinase domain (TKD) mark another class of gain-of-function *FLT3* mutations^4^. Both, ITD and TKD mutations, lead to a constitutive activation of downstream signaling pathways such as the ERK and STAT5 pathway^3^,^4^. With the technical improvement of sequencing methods over recent years the number of novel identified *FLT3* mutations is increasing. However, the evaluation of functional relevance remains difficult, since the mutation’s position in a mutational hotspot or in an important functional receptor domain alone cannot predict its oncogenic potential^5^. In this study we functionally characterized a novel frameshift deletion mutation in the juxtamembrane region (JM) of *FLT3* found in a relapsed patient with AML. We investigated the functional properties of the truncated FLT3 receptor since truncated variants of other receptors have been shown to promote hematopoietic malignancies^6^,^7^,^8^,^9^.

## Materials and Methods

### *FLT3* mutation analysis

A patient was referred to our hospital for treatment of AML relapse. Mononuclear cells were isolated from bone marrow aspirates and conventional cytogenetic and mutational analyses were performed in accordance with described protocols^10^. Sequencing of the *FLT3* gene was performed using Sanger sequencing. The FLT3 variant was described according to the guidelines of the Human Genome Variation Society (HGVS) (http://varnomen.hgvs.org). Mutations in further AML-related genes were analyzed using a targeted, multiplexed amplicon resequencing approach as previously described^10^. The experimental protocols were approved by the Institutional Review Board of the Department of Internal Medicine III, University Hospital Grosshadern, Ludwig-Maximilians-University (LMU), Munich, Germany and written informed consent was obtained in accordance with the Declaration of Helsinki. The methods were performed in accordance with the approved guidelines.

### Cell culture and reagents

The Ba/F3, Hek-293T, and WEHI-3B cell lines were obtained from DSMZ (Braunschweig, Germany), the U2OS cell line from ATCC (Wesel, Germany) and cultured according to the supplier’s recommendation. The retroviral packaging cell line Phoenix eco was purchased from Orbigen (San Diego, CA, USA). Recombinant human FLT3 ligand (FL) was obtained from PromoKine (Heidelberg, Germany), recombinant murine IL-3 from ImmunoTools (Friesoythe, Germany) and AC220 was obtained form Selleck Chemicals (Houston, TX, USA).

### Generation of cell lines

The *FLT3* p.Q569Vfs*2 and the FLAG *FLT3* p.Q569Vfs*2 cDNA were synthesized by GENEART (Life Technologies, Regensburg, Germany) and subcloned into the MSCV-IRES-eYFP retroviral expression vector. The empty vector, MSCV-IRES-eGFP-*FLT3*-WT, and MSCV-IRES-eGFP-*FLT3*-W51 have been described previously^11^. For the stable transduction of Ba/F3 cells the retroviral supernatant of Phoenix eco cells was used^11^. Stable expression of the receptor was confirmed by real time-PCR, Western blot, immunoprecipitation and flow cytometry as described elsewhere^11^,^12^. The following antibodies were used as recommended by the manufacturer: anti FLAG (M2), alpha-Tubulin (T6199), goat anti rabbit secondary antibody from Sigma-Aldrich (St. Louis, MO, USA), FLT3 (SF1.340, S-18), phospho-tyrosine (PY99), goat anti mouse and goat anti rat secondary antibodies from Santa Cruz Biotechnology (CA, USA), ERK and phospho-ERK (Thr202/Tyr204) from Cell Signaling Technology (Danvers, MA, USA). The FLT3-antibody (4B12) was provided by Dr. Elisabeth Kremmer (Helmholtz Center Munich).

### Cell proliferation assay

4 × 10^4^/mL Ba/F3 cells were cultured in the presence or absence of 10 ng IL-3 and 50 ng FL for 72 hours. Viable cells were counted by trypan blue exclusion using the cell viability analyzer Vi-CELL AS (Beckman Coulter, Krefeld, Germany)^11^.

### FL binding assay

Binding of the FL was analyzed using the Fluorokine biotinylated human FL kit (NFFK0) and streptavidin-allophycocyanin (APC) (F0050) (R&D Systems, Minneapolis, MN, USA) according to the supplier’s recommendations. As a negative staining control cells were stained with biotinylated soybean trypsin inhibitor. To confirm staining specificity cells were incubated with unbiotinylated FL in parallel.

### Immunofluorescent staining

U2OS cells were transiently transfected using the PolyFect transfection reagent (QIAGEN, Hilden, Germany). 50,000 cells were seeded on coverslips one day prior transfection. For transfection 1.5 μg plasmid DNA, 5 μL of transfection reagent and Opti-MEM Reduced Serum Medium (Thermo Fisher Scientific, Braunschweig, Germany) were used. The medium was changed six hours later. Glycoconjugates were stained 48 hours post-transfection using anti-wheat germ agglutinin (WGA)-488 fluorescein conjugate (1:1000; Life Technologies, Carlsbad, CA, USA) for 10 minutes. Thereafter cells were fixed on ice for 10 minutes using pre-cooled methanol and blocked for 1 hour with 2% BSA in DPBS. Cells were then incubated with monoclonal mouse or polyclonal rabbit FLT3 (SF1.340, S-18) antibody (1:200; Santa Cruz Biotechnology) for 1 hour, followed by washing with DPBS-T (0.1% Tween 20; Carl Roth, Karlsruhe, Germany). Secondary antibody incubation was performed for 1 hour with anti-mouse IgG (H + L), F (ab’) 2 fragment Alexa Fluor 594 Conjugate (1:500; Cell Signaling Technology). For counterstaining 1 μg/mL 4’,6-diamidino-2-phenylindole (DAPI) was used (Sigma-Aldrich), followed by mounting. Specimens were finally analyzed utilizing a confocal fluorescence laser scanning system (TCS SP5 II; Leica, Wetzlar, Germany). For image acquisition and processing we used the LAS AF Lite Software (Leica).

## Results

### The FLT3 mutation (p.Q569Vfs*2), present only at relapse, results in a truncated FLT3 receptor lacking essential parts for autophosphorylation

**Figure 1:**
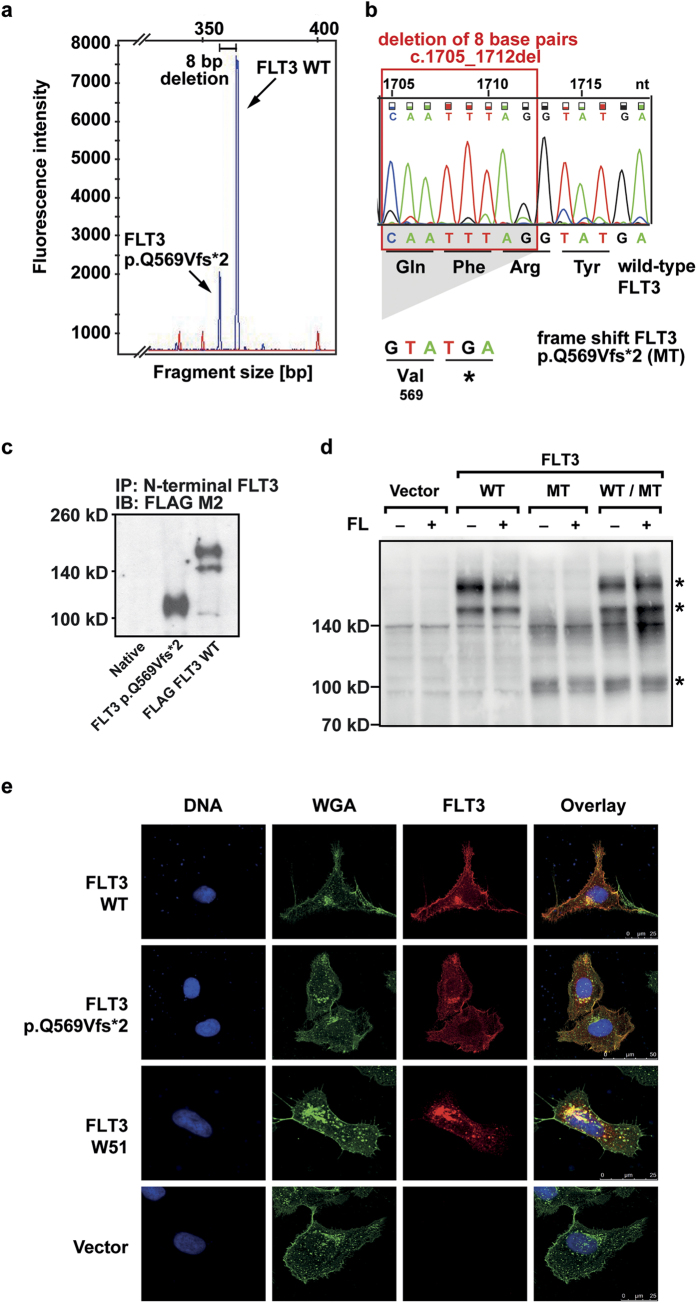
Identification of the mutation in the patient sample and expression of the truncated FLT3 p.Q569Vfs*2 receptor. (**a**) Blast cells were isolated from the bone marrow of a relapsed AML patient. mRNA was isolated and reverse transcribed. The *FLT3* cDNA was amplified and fragment analysis was performed. Arrows indicate fragments for *FLT3* WT and *FLT3* p.Q569Vfs*2. The peaks differ by eight base pairs in their fragment size. (**b**) Sanger sequencing revealed a deletion of eight nucleotides, leading to a frameshift and a premature stop codon within the *FLT3* gene. The chromatogram is shown for the wild-type *FLT3*, nucleotide triplets and the corresponding amino acids are shown for the *FLT3* WT and the frameshift mutation *FLT3* p.Q569Vfs*2 sequence. (**c**) Phoenix eco cells were transfected with FLAG-tagged *FLT3* WT and FLAG-tagged *FLT3* p.Q569Vfs*2. After cell lysis the FLT3 protein was immunoprecipitated from whole cell lysates with an N-terminal FLT3 antibody (SF1.340). After blotting the FLAG-tagged FLT3 was detected with an FLAG M2 antibody. One representative experiment is shown. The blot was cropped to improve the clarity of the image. (**d**) Ba/F3 cells stably expressing the indicated constructs. After cell lysis the FLT3 protein was detected with an N-terminal FLT3 antibody (4B12). One representative experiment is shown. FLT3 bands are indicated by asterisks. The blot was cropped to improve the clarity of the image (MT = FLT3 p.Q569Vfs*2). (**e**) Immunofluorescence of FLT3 (red), glycoconjugates (green) and counterstaining of DNA (blue) in transiently transfected U2OS cells. One representative image of each construct is shown.

The *FLT3* mutation (FLT3 p.Q569Vfs*2) was found during routine diagnostics in a patient with relapsed AML and 15% blasts in the bone marrow (Supplemental Table S1). Fragment size analysis from cDNA showed the *FLT3* wild-type (WT) peak and an additional smaller and shorter fragment (Fig. 1a), indicating a smaller proportion of cells with an alternative *FLT3* transcript. The allele frequency of the detected mutation could not be determined as there was no patient gDNA material available. At the time point of first diagnosis, this fragment was not present (Supplemental Table S1). At the time point of initial diagnosis, the major leukemic clone carried an *IDH2* mutation with an allele frequency of 20.4%. *NPM1c* was present only in a subfraction (7.2% allele frequency) and *FLT3-*ITD/TKD mutations were undetectable (Supplemental Table S1). By Sanger sequencing a deletion in the *FLT3* gene of eight base pairs leading to a frameshift followed by a premature stop codon was identified (Fig. 1b). The mutant is predicted to result in a truncated FLT3 protein, consisting of 570 amino acids and lacking the intracellular parts essential for autophosphorylation of the receptor and downstream signal transduction (Fig. S1). Due to lack of adequate patient material, it was not possible to finally prove the expression of the truncated protein in the patient’s bone marrow. To characterize the functional consequences of the deletion mutation we thus generated Ba/F3 cells stably expressing *FLT3* WT and *FLT3* p.Q569Vfs*2. In Western Blot analysis we observed a band of lower molecular weight (110 kD) in *FLT3* p.Q569Vfs*2-expressing cells in comparison to the WT receptor (140/160 kDa), confirming the expression of a truncated protein (Fig. 1c,d, the full-length blots are shown in supplemental Fig. S4a–c).

### The loss of function FLT3 p.Q569Vfs*2 mutant suppresses FLT3 WT in a dominant negative manner by forming heterodimers with the WT FLT3 receptor

**Figure 2:**
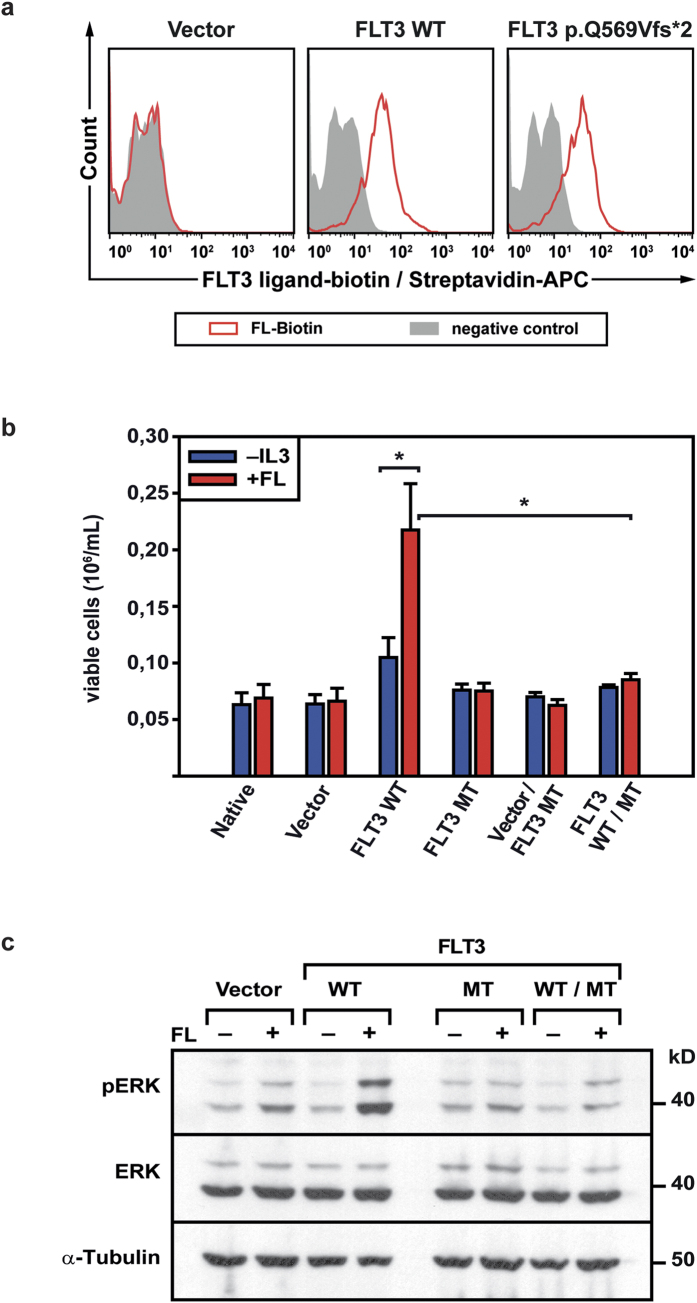
FL binding by the truncated FLT3 p.Q569Vfs*2 receptor, downstream signaling pathways, and proliferation of Ba/F3 cells. (**a**) Ba/F3 cells stably expressing the indicated constructs were incubated with biotinylated human FL. Receptor bound biotinylated FL was detected with streptavidin-APC using flow cytometry. As a negative control biotinylated soybean trypsin inhibitor was used. One out of at least three independent experiments is shown. (**b**) Ba/F3 cells stably expressing the indicated constructs were seeded at a density of 4 × 10^4^/mL in the presence or absence of 50 ng FL. Viable cells were counted by trypan blue exclusion after 72 hours. Shown are mean values ± SEM of at least three independent experiments; *p < 0.05 (MT = FLT3 p.Q569Vfs*2). (**c**) Ba/F3 cells stably expressing the empty vector, *FLT3* WT, *FLT3* p.Q569Vfs*2 alone or both *FLT3* WT and *FLT3* p.Q569Vfs*2 were starved for 24 hours in cell culture media containing 0.3% fetal calf serum. Cells were left untreated (−) or stimulated (+) with 100 ng/mL FL for 60 minutes prior to cell lysis. Phosphorylation of ERK was analyzed by western blot. One representative experiment is shown. The blot was first incubated with phospho ERK antibody, stripped and reblotted with ERK and α-Tubulin antibody. The blot was cropped to improve the clarity of the image (MT = FLT3 p.Q569Vfs*2).

FLT3 p.Q569Vfs*2 receptor expression on the cell surface was confirmed by flow cytometry and immunofluorescent staining (Fig. 1e, Supplemental Fig. S2) and binding of FL by FL binding assays with biotinylated FL (Fig. 2a). After adding of unbiotinylated FL to the sample, the binding capacity for biotinylated FL decreased in a dose dependent manner indicating the specificity of the FL binding assay (Supplemental Fig. S3). By immunoprecipitation the truncated FLT3 protein in double-transfected Hek-293T cells, and immunoblot against a C-terminal FLT3 epitope, heterodimerisation of the truncated and the wildtype FLT3 receptor became detectable (Fig. S5b). In contrast to *FLT3* WT, *FLT3* p.Q569Vfs*2-expressing Ba/F3 cells did not proliferate after FL stimulation (Fig. 2b). Furthermore, coexpression of WT and mutant FLT3 receptor also abolished the WT receptor’s proliferative effect on Ba/F3 cells when stimulated with FL (Fig. 2b). This observation was confirmed by analyzing the downstream signaling pathway. Stimulation of *FLT3* WT-expressing cells with FL led to strong FLT3 and subsequent ERK phosphorylation which were absent in *FLT3* p.Q569Vfs*2-expressing and *FLT3* p.Q569Vfs*2/*FLT3* WT-coexpressing cells, respectively (Fig. 2c, the full-length blots are shown in supplemental Fig. S4d,e and Fig. S5c,d).

In order to address a possible growth advantage of the *FLT3* p.Q569Vfs*2-expressing cells, we performed proliferation assays in Ba/F3 cells treated with the highly potent FLT3 inhibitor AC220 in two different concentrations. Neither the *FLT3* p.Q569Vfs*2 cells nor the double-transduced cells had a growth advantage under the chosen conditions (see supplemental Fig. S5a).

## Discussion

AML-specific mutations within the *FLT3* gene have been of high interest and were studied in detail over the past decades. In contrast to the highly prevalent activating mutations the impact of receptor truncating mutations in *FLT3* remains largely unknown, especially regarding their proliferative activity. To our knowledge, this is the first study to functionally characterize a patient derived loss-of-function *FLT3* mutation resulting in a dominant negative effect on the WT receptor. Our analysis demonstrated that the truncated FLT3 receptor lack both TKDs that are crucial for signal transduction, thus being unable to activate ERK phosphorylation and proliferation upon ligand binding. These effects were dominant negative on FLT3 WT in cells coexpressing both *FLT3* WT and p.Q569Vfs*2.

In analogy, kinase-negative or truncated EGF receptors can exert a dominant negative effect on WT receptors by inhibiting the tyrosine kinase activity and suppressing the mitogenic response of WT receptors through heterodimerisation^13^,^14^. However, the receptor truncation does not always imply a loss of function. Alterations of the *CSF3R* gene, which lead to a truncated cytoplasmic tail, showed an activating and hyperresponsive phenotype and have been linked to the development of AML, chronic myeloid and neutrophilic leukemia^6^,^7^. Similary, truncations in the extracellular domain of the TrkA receptor (“Delta TrkA”) constitutively activate the receptor in fibroblast and epithelial cell lines^8^,^9^.

The loss-of-function phenotype of *FLT3* p.Q569Vfs*2 suggests that the patient’s AML blasts proliferate independently of FLT3 kinase activity. We cannot exclude that *FLT3* p.Q569Vfs*2 is a passenger mutation in a mutational hotspot region of the *FLT3* gene and does not influence leukemogenesis. However, the same somatic deletion mutation has been reported recently as a somatic and recurrent mutation, detected in two out of 6843 unselected AML patients resulting in a frequency of 0.03%^15^. It is tempting to speculate that a leukemic cell may benefit from truncating *FLT3* mutations through escape from the FLT3 receptor mediated growth regulation. Functional characterization of *FLT3* mutations nevertheless is of major importance to identify driver mutations, validate therapeutic targets and analyze the mechanisms of receptor interaction and activation. Our study shows that mutations of *FLT3* do not always accompany a gain of function and functional relevance has to be investigated on an individual basis.

## Additional Information

**How to cite this article**: Sandhöfer, N. *et al*. The new and recurrent FLT3 juxtamembrane deletion mutation shows a dominant negative effect on the wild-type FLT3 receptor. *Sci. Rep.* **6**, 28032; doi: 10.1038/srep28032 (2016).

## Supplementary Material

Supplementary Information
