# Supplementary material for: The new and recurrent FLT3 juxtamembrane deletion mutation shows a dominant negative effect on the wild-type FLT3 receptor
Source: Sci Rep. 2016 Jun 27;6:28032. doi: 10.1038/srep28032 (PMC4921855; doi:10.1038/srep28032)
Supplement: Supplementary Information [file srep28032-s1.pdf]

Supplementary information

The new and recurrent FLT3 juxtamembrane deletion mutation shows a dominant negative effect on the wild-type FLT3 receptor

Nadine Sandhöfer, Julia Bauer, Katrin Reiter, Annika Dufour, Maja Rothenberg, Nikola P. Konstandin, Evelyn Zellmeier, Belay Tizazu, Philipp A. Greif, Klaus H. Metzeler, Wolfgang Hiddemann, Harald Polzer and Karsten Spiekermann

Supplemental Tables:

- Supplemental Table S1: Overview about available material and performed analyses.

Supplemental Figures:

- Supplemental Figure S1: Schematic representation of the juxtamembrane deletion mutant FLT3 receptor in comparison to the WT receptor.
- Supplemental Figure S2: Cell surface expression of FLT3 constructs.
- Supplemental Figure S3: Binding capacity for biotinylated FLT3 ligand.
- Supplemental Figure S4: Full-length immuno blots of figure 1 and 2.
- Supplemental Figure S5: Dominant negative effect of FLT3 p.Q569Vfs*2 and its heterodimerisation with FLT3 WT

Figure S1

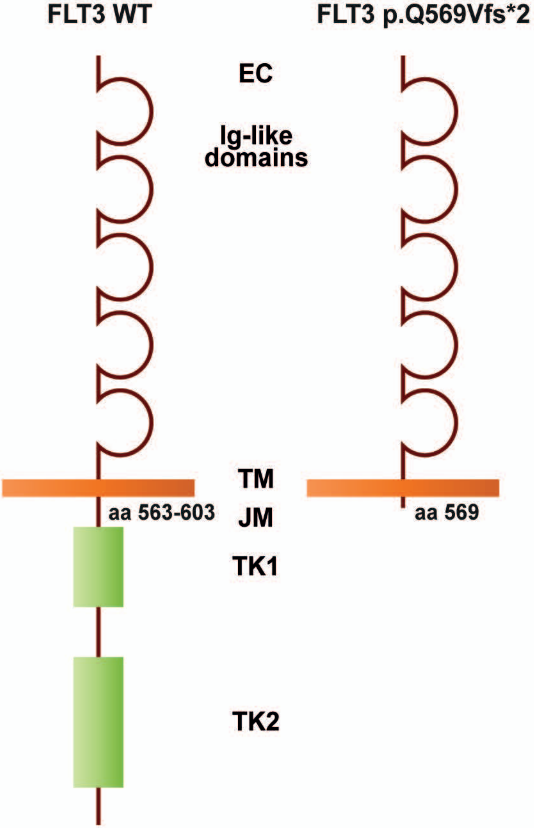

Figure S1: Schematic representation of the juxtamembrane deletion mutant FLT3 receptor in comparison to the FLT3 WT receptor. The mutated receptor lacks a large proportion of the juxtamembrane domain as well as other intracellular domains, especially the tyrosine kinase domains 1/2.

aa: amino acid, EC: extracellular domain, Ig-lke domain: Immunglobuline-like domain, TM: transmembrane domain, JM: juxtamembrane domain, TK1/2: tyrosine kinase domain 1/2.

Figure S2

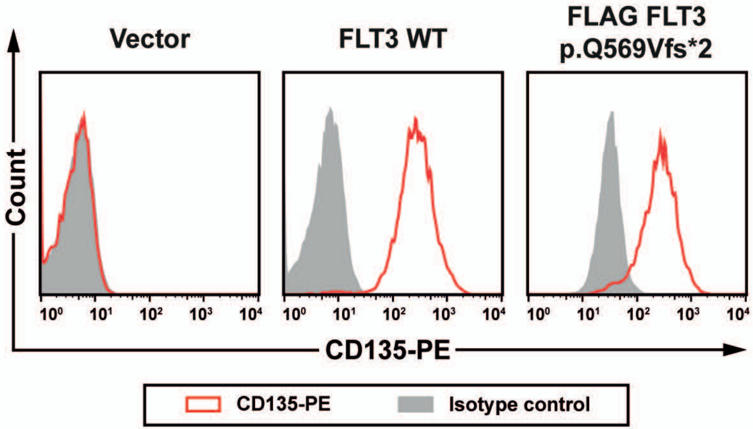

Figure S2: Cell surface expression of the FLT3 constructs. Ba/F3 cells stably expressing the indicated constructs were stained with CD135 antibody (red) or isotype control (grey) and analyzed by flow cytometry. One representative example is shown.

Figure S3

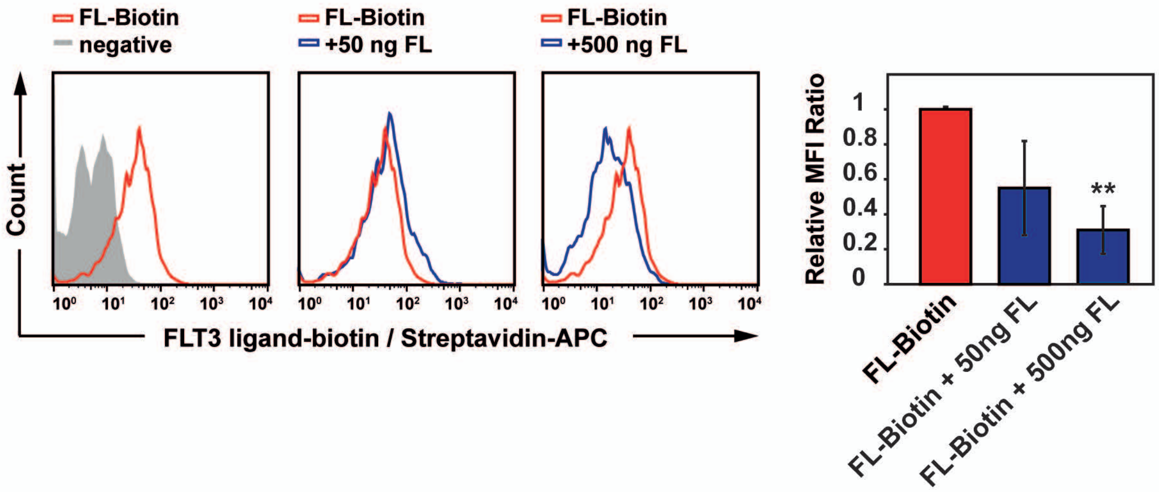

Figure S3: Binding capacity for biotinylated FLT3 ligand. Ba/F3 cells stably expressing FLT3 p.Q569Vfs*2 were incubated with biotinylated human FLT3 ligand (FL). Receptor bound biotinylated FL was detected with streptavidin-APC using flow cytometry. As a negative control biotinylated soybean trypsin inhibitor was used. Different amounts of unbiotinylated FL (50 ng; 500 ng) were added to determine the staining specificity. One representative example is shown (left). Binding of biotinylted FL to FLT3 p.Q569Vfs*2 without addition of unbiotinylated FL is set as 1. Shown are mean values of the relative mean fluorescence intensity ratios (MFI) ± SEM of at least three independent experiments (right); ** $p<0.01$.

Figure S4

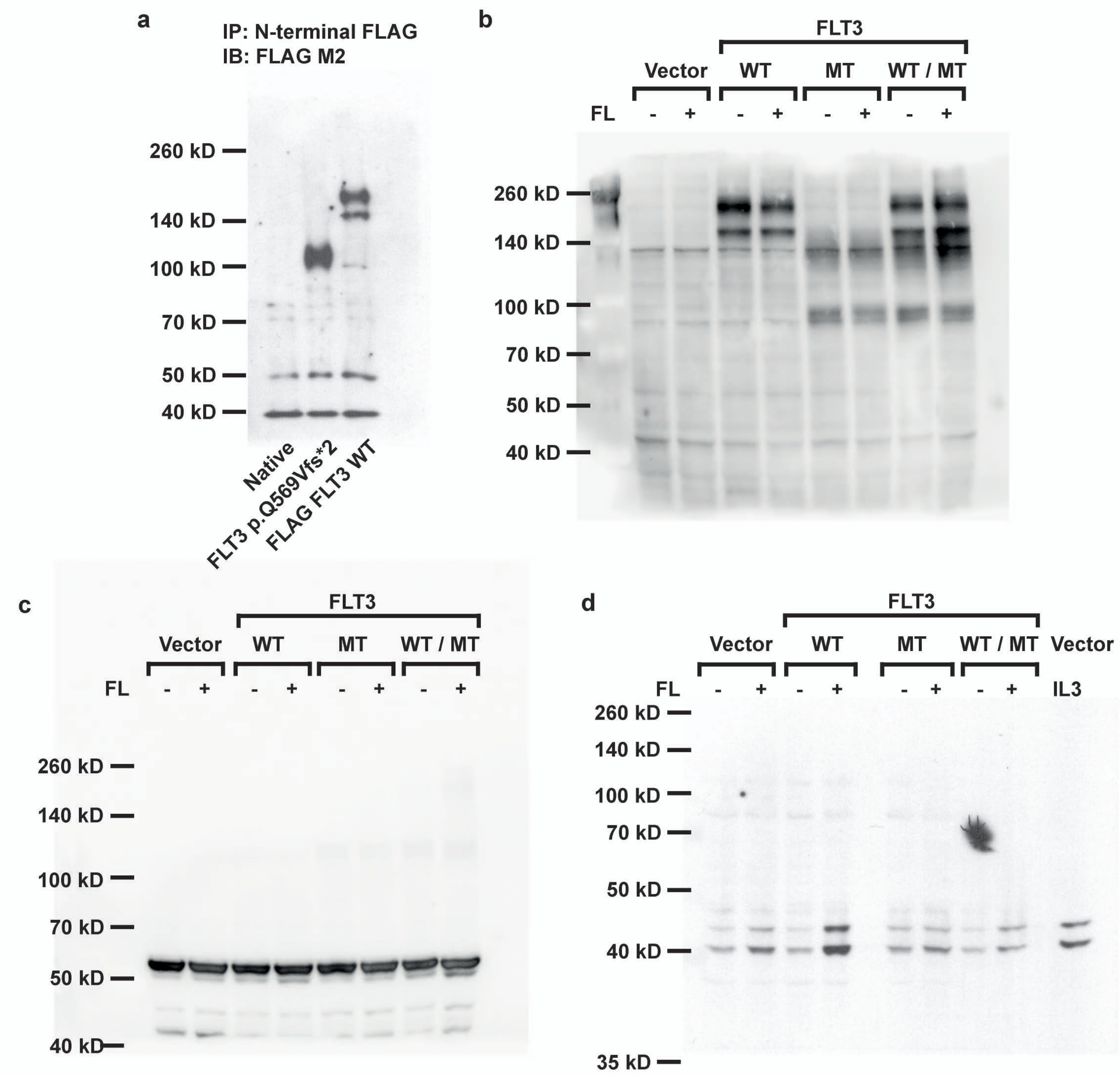

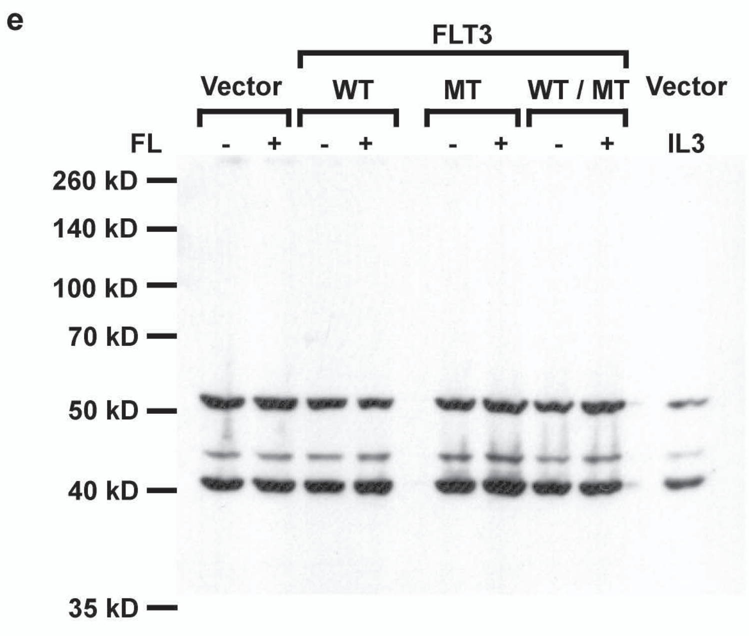

Figure S4: Full-lenght immuno blots of Figure 1 and 2. (a) Phoenix Eco cells were transfected with indicated constructs and expression analysed by immuno blot. It is the flull-lenght immuno blot of Figure 1c. (b) Ba/F3 cells stably expressing the indicated constructs were analyzed by immuno blot. After cell lysis the FLT3 protein was detected with an FLT3 antibody. It is the full-length immuno blot of Figure 1d. (c) α-Tubulin was detected in the whole cell lysates of stably transduced Ba/F3 cells as loading control. It is the full-lenth immuno blot of Figure 1d. (d) Full-length immuno blot of Figure 2c. Phosphorylation of ERK was analyzed of stably transduced Ba/F3 cells. (e) First ERK and then α-Tubulin were analyzed on the blot. It is the full-length immuno blot of Figure 2c.
(MT = FLT3 p.Q569Vfs*2)

Figure S5

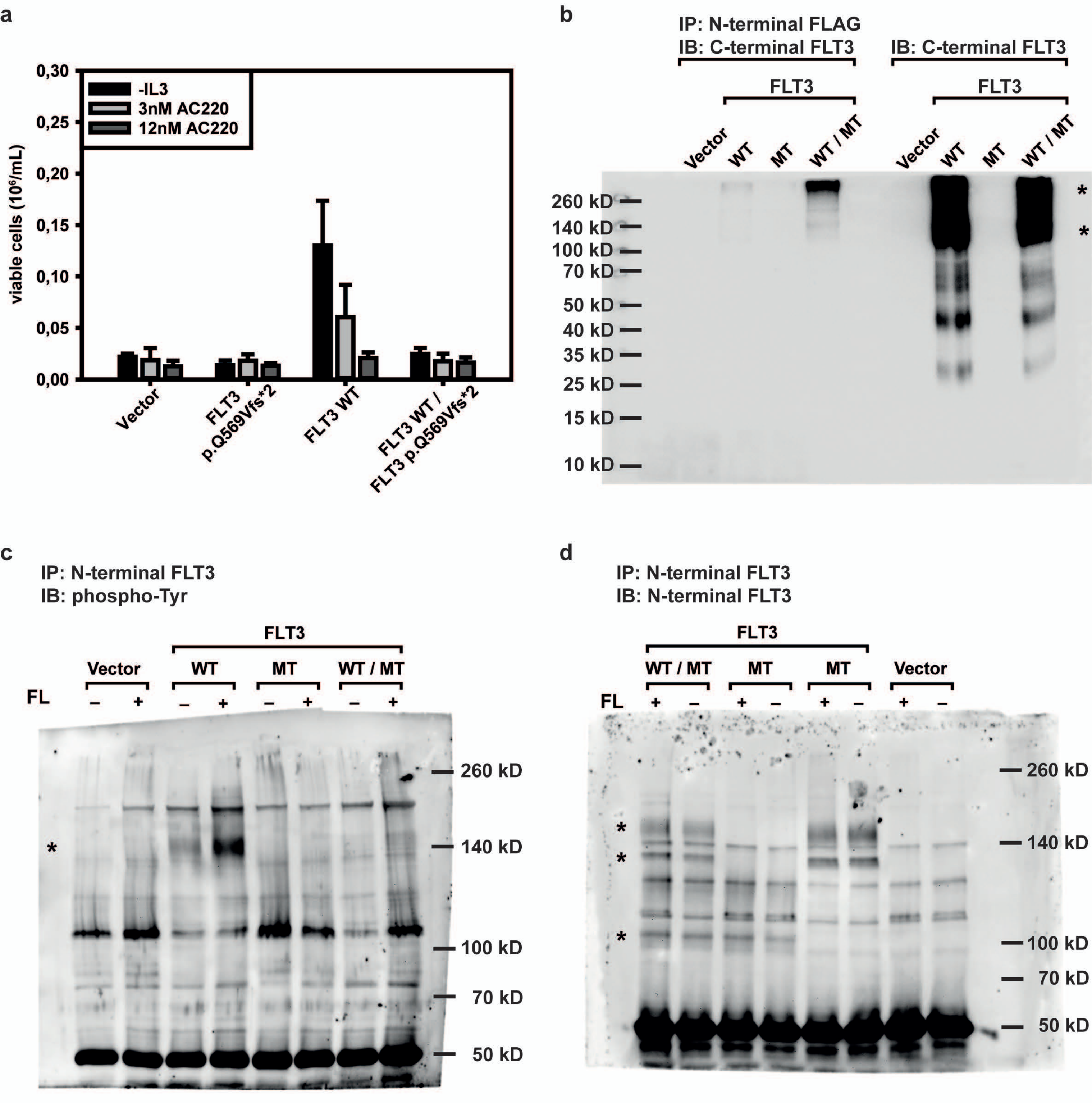

Figure S5: Dominant negative effect of FLT3 p.Q569Vfs*2 and its heterodimerisation with FLT3 WT. (a) Ba/F3 cells stably expressing the indicated constructs were seeded at a density of 4×10⁴/mL in the absence of cytokines and indicated concentrations of AC220. Viable cells were counted by trypan blue exclusion after 72 hours. Shown are mean values ± SEM of three independent experiments. (b) HEK 293T were transfected with indicated consructs. After lysis FLT3 p.Q569Vfs*2 was immunoprecipitated via its FLAG-tag from whole cell lysates. SDS-gel was run without reducing agents and in MOPS buffer. After blotting the FLT3 WT was detected with a C-terminal FLT3 antibody (* indicating FLT3 dimer and monomer; MT = FLT3 p.Q569Vfs*2). (c,d) From whole cell lysates of stable Ba/F3 cells the FLT3 protein was immunoprecipitated with an N-terminal FLT3 antibody (SF1.340) (* indicating FLT3 specific bands; MT = FLT3 p.Q569Vfs*2). After blotting the phosphorylation status of FLT3 was detected by a pTyr antibody (c) and total FLT3 protein with a different N-terminal FLT3 antibody (4B12) (d).
